# 1918. A Year's Work in the Hospitals and Medical Charities of London

**Published:** 1919-06-21

**Authors:** 


### 1918.

### A YEAR'S WORK IN THE HOSPITALS AND MEDICAL CHARITIES OF LONDON.

### ST. MARYLEBONE AND WEST CENTRAL DISTRICT.

Comprising St. Marylebone, St. John's Wood, Bloomsbury, Holborn, &c.

| No. of Beds. | No. of Beds Daily Occupied. | HOSPITALS. | In-patients. | Out-patient Attendances. | Total Expenditure. | Income. | | | Total Income. | Legacies not included in preceding column. |
|---|---|---|---|---|---|---|---|---|---|---|
| | | | | | | Charitable. | Proprietary. | Patients' Payments. | | |
| | | | | | £ | £ | £ | £ | £ | £ |
| 87 | 72 | French ... ... ... ... | 878 | 19,553 | 9,707 | 7,056 | 2,193 | 3,327 | 12,576 | 1,626 |
| 85 | 63 | Italian ... ... ... ... | 739 | 5,291 | 8,418 | 2,806 | 2,305 | 4,298 | 9,409 | 50 |
| 165 | 157 | London Homœopathic ... ... | 1,776 | 48,482 | 21,980 | 7,432 | 5,548 | 9,684 | 22,664 | 7,480 |
| 172 | 133 | SS. John and Elizabeth ... ... | 1,171 | ... | 19,679 | 2,475 | 7,369 | 8,319 | 18,163 | 31 |
| 361 | 291 | The Middlesex ... ... ... | 4,412 | 166,278 | 61,123 | 23,852 | 10,796 | 12,715 | 47,363 | 10,174 |
| 68 | 53 | Alexandra, for Children... ... | 38 | 378 | 6,891 | 3,126 | 1,142 | 3,306 | 7,574 | 200 |
| 204 | 191 | Hospital for Sick Children ... | 2,567 | 80,996 | 30,586 | 15,166 | 7,969 | 2,770 | 25,905 | 14,593 |
| 24 | 21 | S. Monica's, for Children ... | 112 | ... | 1,764 | 941 | 145 | 420 | 1,506 | 700 |
| 69 | 58 | Queen Charlotte's Lying-In ... | 1,649 | 16,190 | 13,527 | 9,487 | 1,244 | 2,592 | 13,323 | 55 |
| 71 | 66 | Elizabeth Garrett Anderson Hospital ... ... ... | 1,189 | 34,166 | 14,946 | 3,826 | 2,485 | 3,994 | 10,305 | 3,860 |
| 70 | 53 | Samaritan Free ... ... ... | 990 | 12,709 | 10,052 | 5,499 | 811 | ... | 6,310 | 3,686 |
| 167 | 157 | National for the Paralysed, &c.... | 735 | 51,140 | 30,229 | 10,338 | 3,270 | 14,642 | 28,250 | 4,117 |
| 86 | 80 | Hospital for Epilepsy, &c. ... | 474 | 21,191 | 10,080 | 5,488 | 292 | 4,314 | 10,094 | 100 |
| 102 | 89 | West End, for Epilepsy, &c. ... | 450 | 36,133 | 12,612 | 4,731 | 1,953 | 5,717 | 12,401 | 275 |
| 40 | 23 | Central London Ophthalmic ... | 386 | 30,256 | 4,243 | 3,704 | 342 | ... | 4,046 | ... |
| 16 | 13 | Western Ophthalmic ... ... | 346 | 21,026 | 2,401 | 2,723 | 277 | 483 | 3,483 | 314 |
| 200 | 160 | Royal National Orthopædic ... | 981 | 48,728 | 22,197 | 8,454 | 1,734 | 12,246 | 22,434 | 376 |
| 38 | 29 | Florence Nightingale Home ... | 425 | ... | 5,736 | 2,523 | 373 | 2,737 | 5,633 | 350 |
| 49 | 47 | The Middlesex Cancer ... ... | 143 | 605 | 7,250 | 2,430 | 3,121 | ... | 5,551 | 950 |
| 28 | 9 | Metropolitan Ear, &c. ... ... | 272 | 9,866 | 2,436 | 751 | 1 | 1,439 | 2,191 | ... |
| 2,102 | 1,765 | | 19,773 | 602,988 | 295,857 | 122,808 | 53,370 | 93,003 | 269,181 | 48,937 |
| | | DISPENSARIES. | | | | | | | | |
| ... | ... | London Medical Mission ... | ... | 5,330 | 1,033 | 827 | 476 | 129 | 1,432 | 165 |
| ... | ... | Margaret Street, for Consumption | ... | 8,480 | 945 | 499 | 571 | ... | 1,070 | 140 |
| ... | ... | St. John's Wood Provident ... | ... | 16,171 | 465 | 335 | 27 | 135 | 497 | 25 |
| ... | ... | St. Marylebone General ... ... | ... | 11,656 | 1,466 | 531 | 233 | 410 | 1,174 | ... |
| ... | ... | Western General ... ... ... | ... | 10,508 | 1,021 | 571 | 169 | 20 | 760 | 514 |
| 2,102 | 1,765 | | 19,733 | 655,133 | 300,787 | 125,571 | 54,846 | 93,697 | 274,114 | 49,781 |

### WESTMINSTER DISTRICT.—Comprising Westminster City and Liberties.

| No. of Beds. | No. of Beds Daily Occupied. | HOSPITALS. | In-patients. | Out-patient Attendances. | Total Expenditure. | Charitable. | Proprietary. | Patients' Payments. | Total Income. | Legacies not included in preceding column. |
|---|---|---|---|---|---|---|---|---|---|---|
| | | | | | £ | £ | £ | £ | £ | £ |
| 300 | 250 | Charing Cross ... ... ... | 3,204 | 81,212 | 45,241 | 34,329 | 4,495 | 12,766 | 51,590 | 2,817 |
| 218 | 192 | Westminster ... ... ... | 1,799 | 70,633 | 36,094 | 27,098 | 5,323 | 9,493 | 41,914 | 4,898 |
| 160 | 158 | Ventnor, for Consumption ... | 646 | ... | 19,784 | 5,965 | 3,652 | 9,815 | 19,432 | 1,360 |
| 37 | 20 | Grosvenor, for Women & Children | 335 | 6,064 | 3,745 | 1,904 | 541 | 670 | 3,115 | 346 |
| 68 | 59 | Hospital for Women ... ... | 1,020 | 12,106 | 9,029 | 6,180 | 787 | 1,706 | 8,673 | 682 |
| 42 | 37 | National, for Diseases of Heart... | 214 | 19,614 | 6,694 | 2,509 | 1,221 | 2,818 | 6,548 | 12,330 |
| 40 | 30 | Male Lock ... ... ... | 351 | 40,441 | 7,998 | 1,157 | 5 | 6,150 | 7,312 | 50 |
| 40 | 33 | Royal Westminster Ophthalmic... | 531 | 26,418 | 4,906 | 2,712 | 1,391 | 855 | 4,958 | 1,525 |
| ... | ... | Royal Dental ... ... ... | ... | 26,420 | 4,339 | 4,541 | 1,156 | 402 | 6,099 | 275 |
| 32 | 22 | St. Peter's (Stone) ... ... | 380 | 31,755 | 7,022 | 4,112 | 577 | 2,492 | 7,181 | 294 |
| 50 | 37 | Infants' Hospital, Vincent Square | 352 | 3,067 | 4,309 | 3,768 | 85 | ... | 3,853 | 100 |
| 40 | 18 | St. John's, for Skin Diseases ... | 148 | 37,668 | 5,641 | 2,843 | 13 | 3,013 | 5,869 | ... |
| 57 | 37 | Throat Hospital, Golden Square | 1,067 | 46,665 | 8,190 | 4,646 | 379 | 5,006 | 10,031 | 8,946 |
| 1,084 | 893 | | 10,047 | 401,763 | 162,992 | 101,764 | 19,625 | 55,186 | 176,575 | 33,623 |
| | | DISPENSARIES. | | | | | | | | |
| ... | ... | Public ... ... ... ... ... | ... | 10,278 | 758 | 336 | 209 | ... | 545 | 75 |
| ... | ... | St. George's, Hanover Square ... | ... | 1,797 | 406 | 231 | 85 | 86 | 402 | ... |
| ... | ... | Western ... ... ... ... | ... | 14,052 | 1,338 | 212 | 413 | 557 | 1,182 | 25 |
| ... | ... | Westminster General ... ... | ... | 13,360 | 932 | 361 | 442 | 89 | 892 | ... |
| 1,084 | 893 | | 10,047 | 441,250 | 166,426 | 102,904 | 20,774 | 55,918 | 179,596 | 33,723 |

### CITY AND EAST CENTRAL DISTRICT.

Comprising the City, St. Luke's, Shoreditch, Finsbury, and Clerkenwell.

| No. of Beds. | No. of Beds Daily Occupied. | Hospitals. | In-patients. | Out-patient Attendances. | Total Expenditure. | Income. | | | Total Income. | Legacies not included in preceding column. |
|---|---|---|---|---|---|---|---|---|---|---|
| | | | | | | Charitable. | Proprietary. | Patients' Payments. | | |
| | | | | | £ | £ | £ | £ | £ | £ |
| 405 | 343 | Metropolitan ... ... ... | 3,198 | 80,972 | 45,366 | 16,298 | 1,224 | 25,987 | 43,509 | 961 |
| 200 | 174 | Royal Free ... ... ... | 2,547 | 120,589 | 38,021 | 10,646 | 5,608 | 5,051 | 21,305 | 8,369 |
| 689 | 505 | St. Bartholomew's ... ... | 6,612 | 229,750 | 124,399 | 13,865 | 81,379 | 19,452 | 114,696 | 2,908 |
| 110 | 86 | Royal, for Diseases of the Chest. | 793 | 30,019 | 14,410 | 5,030 | 620 | 10,626 | 16,276 | 100 |
| 134 | 110 | Queen's, for Children ... ... | 1,481 | 107,075 | 23,783 | 19,966 | 1,138 | 1,040 | 22,144 | 610 |
| 61 | 50 | City of London Maternity ... | 1,325 | 4,657 | 9,860 | 4,409 | 2,798 | 1,599 | 8,806 | ... |
| 11 | 9 | Jewish Maternity Hospital ... | 275 | 252 | 2,251 | 1,122 | 439 | 564 | 2,125 | ... |
| 56 | 46 | St. Mark's, for Fistula ... ... | 705 | 4,258 | 8,108 | 4,252 | 1,390 | 406 | 6,048 | 552 |
| 138 | 113 | Royal London Ophthalmic ... | 2,333 | 98,186 | 20,035 | 11,482 | 2,291 | 3,440 | 17,213 | 2,573 |
| 43 | 35 | Central London Throat and Ear. | 505 | 40,605 | 6,840 | 1,911 | 402 | 4,999 | 7,312 | 702 |
| 1,847 | 1,471 | | 19,774 | 716,363 | 293,073 | 88,981 | 97,289 | 73,164 | 259,434 | 16,775 |
| | | **Dispensaries.** | | | | | | | | |
| ... | ... | Billingsgate Medical Mission ... | ... | 8,471 | 743 | 510 | 1 | 96 | 607 | ... |
| ... | ... | City ... ... ... ... | ... | 12,468 | 847 | 781 | 103 | ... | 884 | 500 |
| ... | ... | Farringdon General ... ... | ... | 4,977 | 567 | 380 | 15 | 168 | 563 | ... |
| ... | ... | Finsbury ... ... ... ... | ... | 29,182 | 1,419 | 942 | 201 | 316 | 1,459 | ... |
| ... | ... | Metropolitan ... ... ... | ... | 2,528 | 666 | 255 | 255 | 86 | 596 | ... |
| ... | ... | Royal General ... ... ... | ... | 6,663 | 855 | 215 | 429 | 46 | 690 | ... |
| 1,847 | 1,471 | | 19,774 | 780,652 | 298,170 | 92,064 | 98,293 | 73,876 | 264,233 | 17,275 |

### ISLINGTON AND NORTH-WEST DISTRICT.

Comprising Islington, Holloway, Highbury, Hampstead, Highgate, St. Pancras, Stoke Newington, Tottenham, &c.

| No. of Beds. | No. of Beds Daily Occupied. | Hospitals. | In-patients. | Out-patient Attendances. | Total Expenditure. | Income. | | | Total Income. | Legacies not included in preceding column. |
|---|---|---|---|---|---|---|---|---|---|---|
| | | | | | | Charitable. | Proprietary. | Patients' Payments. | | |
| | | | | | £ | £ | £ | £ | £ | £ |
| 324 | 298 | Great Northern Central ... ... | 2,834 | 123,487 | 52,429 | 27,569 | 2,427 | 24,067 | 54,063 | 1,305 |
| 137 | 118 | Hampstead General Hospital ... | 1,337 | 30,418 | 20,534 | 10,826 | 1,353 | 5,554 | 17,733 | 375 |
| 120 | 94 | London Temperance ... ... | 1,154 | 68,570 | 14,000 | 5,098 | 2,144 | 2,802 | 10,044 | 748 |
| 125 | 104 | Tottenham (Prince of Wales's)... | 2 019 | 80,341 | 17,017 | 12,697 | 897 | 221 | 13,815 | ... |
| 505 | 460 | University College ... ... | 4,508 | 134,720 | 56,030 | 21,655 | 7,782 | 28,766 | 58,203 | 15,891 |
| 110 | 105 | Mount Vernon, for Consumption | 342 | 21,376 | 15,814 | 3,177 | 4,187 | 7,245 | 14,609 | 1,300 |
| 20 | 19 | Children's Home Hospital, Barnet | 30 | ... | 670 | 458 | 243 | 59 | 760 | ... |
| 160 | 43 | London Fever ... ... ... | 685 | ... | 13,026 | 6,064 | 1,687 | 3,926 | 11,677 | 263 |
| 50 | 68 | Salvation Army Mothers' ... | 969 | ... | 7,035 | 1,331 | 3,394 | 2,688 | 7,413 | ... |
| 25 | 21 | Enfield Cottage ... ... ... | 248 | ... | 1,868 | 1,581 | 160 | 376 | 2,117 | 450 |
| 17 | 14 | Hendon Cottage ... ... ... | 232 | ... | 1,880 | 1,150 | 97 | 905 | 2,152 | ... |
| 23 | 13 | St. Saviour's Hospital ... ... | 131 | ... | 2,812 | 1,016 | 349 | 1,003 | 2,368 | ... |
| 48 | 37 | St. Columba's Hospital ... ... | 148 | ... | 6,852 | 3,895 | 395 | 560 | 4,850 | 1,835 |
| 72 | 55 | Willesden Cottage ... ... | 583 | 1,045 | 5,714 | 1,776 | 193 | 4,924 | 6,893 | ... |
| 30 | 19 | Wood Green Cottage ... ... | 309 | 74 | 2,469 | 1,577 | 83 | 708 | 2,368 | ... |
| 20 | 20 | Santa Claus Home ... ... | 20 | ... | 1,342 | 1,009 | 119 | 108 | 1,236 | ... |
| 26 | 10 | Stoke Newington Home Hospital | 111 | ... | 1,316 | 898 | 379 | 272 | 1,549 | 20 |
| 18 | 17 | Winifred House, Holloway ... | 13 | ... | 1,123 | 542 | 213 | 235 | 990 | 2,449 |
| 20 | 20 | Highgate Convalescent Home ... | 126 | ... | 648 | 548 | 45 | 125 | 718 | ... |
| 86 | 72 | St. Andrew's, Dollis Hill ... | 881 | ... | 8,651 | 1,422 | 449 | 6,815 | 8,686 | ... |
| 1,937 | 1,607 | | 16,680 | 460,031 | 231,230 | 104,289 | 26,596 | 91,359 | 222,244 | 24,136 |
| | | **Dispensaries** | | | | | | | | |
| ... | ... | Child's Hill Provident ... ... | ... | 1,500 | 103 | 18 | 8 | 77 | 103 | ... |
| ... | ... | Hampstead Provident ... ... | ... | 11,529 | 900 | 389 | 103 | 524 | 1,016 | ... |
| ... | ... | Islington ... ... ... ... | ... | 42,536 | 967 | 176 | 82 | 709 | 967 | ... |
| ... | ... | Islington Medical Mission ... | ... | 15,337 | 1,092 | 651 | 487 | 130 | 1,268 | ... |
| ... | ... | St. Pancras and Northern ... | ... | 8,332 | 1,776 | 310 | 164 | 1,236 | 1,710 | 10 |
| ... | ... | Stamford Hill, &c. ... ... | ... | 22,291 | 683 | 420 | 154 | ... | 574 | 20 |
| ,937 | 1,607 | | 16,680 | 559,556 | 236,751 | 106,253 | 27,594 | 94,035 | 227,882 | 24,166 |

### STRATFORD AND EAST-END DISTRICT.

**Comprising Bethnal Green, Tower Hamlets, West Ham, Whitechapel, Hackney, Stepney, Limehouse, Poplar, and the East.**

| No. of Beds. | No. of Beds Daily Occupied. | HOSPITALS. | In-patients | Out-patient Attendances. | Total Expenditure. | Income. Charitable. | Income. Proprietary. | Income. Patients' Payments. | Total Income. | Legacies not included in preceding column. |
|---|---|---|---|---|---|---|---|---|---|---|
| | | | | | £ | £ | £ | £ | £ | £ |
| 933 | 805 | London ... ... ... ... | 16,493 | 356,370 | 217,990 | 183,691 | 41,954 | 28,541 | 254,186 | 27,450 |
| 50 | 39 | Mildmay Mission Hospital ... | 652 | 26,020 | 4,924 | 3,715 | 646 | 631 | 4,992 | ... |
| 29 | 24 | Mildmay Memorial ... ... | 285 | ... | 2,778 | 1,533 | 1,145 | 507 | 3,185 | ... |
| 115 | 83 | Poplar ... ... ... ... | 1,746 | 77,923 | 16,865 | 14,660 | 3,250 | 1,715 | 19,625 | 1,968 |
| 50 | 38 | Walthamstow, &c. ... ... | 504 | 11,893 | 2,982 | 2,550 | 546 | 38 | 3,134 | ... |
| 179 | 164 | Queen Mary's, &c. ... ... | 1,764 | 131,743 | 25,509 | 12,378 | 1,655 | 7,159 | 21,192 | 18 |
| 175 | 161 | City of London for Dis. of the Chest | 918 | 39,508 | 25,095 | 13,417 | 1,308 | 5,221 | 19,946 | 1,729 |
| 130 | 92 | East London, for Children ... | 1,361 | 56,730 | 16,336 | 12,958 | 2,623 | 47 | 15,628 | 857 |
| 43 | 43 | St. Mary's, Plaistow, for Children | 629 | 23,359 | 5,711 | 4,062 | 708 | 96 | 4,866 | 1,421 |
| 33 | 27 | East End Mothers' Home ... | 757 | 14,471 | 3,824 | 2,200 | 1,273 | 844 | 4,317 | ... |
| 20 | 15 | Plaistow Maternity ... ... | 503 | ... | 1,497 | 1,096 | 1 | 394 | 1,491 | ... |
| 12 | 28 | Brentwood Cottage ... ... | 78 | ... | 914 | 427 | 144 | 190 | 761 | ... |
| 26 | 17 | Canning Town Cottage ... ... | 246 | 6,495 | 2,650 | 1,733 | 319 | 389 | 2,441 | ... |
| 17 | 9 | Passmore Edwards Cottage, T'lb'ry | 187 | 2,418 | 905 | 1,038 | 88 | 37 | 1,163 | ... |
| 25 | 13 | East Ham Cottage ... ... | 102 | 14,443 | 1,724 | 1,079 | 546 | 424 | 2,049 | ... |
| 1,837 | 1,558 | | 26,225 | 761,373 | 329,704 | 256,537 | 56,206 | 46,233 | 358,976 | 33,443 |
| | | DISPENSARIES. | | | | | | | | |
| ... | ... | Eastern ... ... ... ... | ... | 17,564 | 686 | 225 | 269 | 302 | 796 | ... |
| ... | ... | London ... ... ... ... | ... | 4,337 | 472 | 86 | 258 | ... | 344 | ... |
| ... | ... | Queen Adelaide's ... ... ... | ... | 8,412 | 604 | 605 | 372 | ... | 977 | ... |
| 1,837 | 1,558 | | 26,225 | 791,686 | 331,466 | 257,453 | 57,105 | 46,535 | 361,093 | 33,443 |

### KENSINGTON AND WEST DISTRICT.

**Comprising Kensington, Paddington, Bayswater, Kilburn, Chelsea, Brompton, Fulham, Hammersmith, Chiswick, Brentford, Acton, Ealing, &c.**

| | | HOSPITALS. | | | £ | £ | £ | £ | £ | £ |
|---|---|---|---|---|---|---|---|---|---|---|
| 341 | 300 | St. George's ... ... ... | 3,932 | 110,693 | 59,591 | 16,898 | 14,663 | 8,689 | 40,250 | 20,222 |
| 302 | 251 | St. Mary's ... ... ... ... | 3,034 | 96,446 | 46,480 | 11,774 | 12,560 | 8,475 | 32,809 | 16,370 |
| ... | ... | West London ... ... ... | ... | ... | ... | ... | ... | ... | ... | ... |
| 483 | 420 | Hospital for Consumption ... | 1,637 | 27,566 | 59,407 | 23,159 | 4,068 | 34,062 | 61,289 | 10,777 |
| 40 | 35 | Belgrave, for Children ... ... | 560 | 33,711 | 6,265 | 3,660 | 1,008 | 370 | 5,038 | 50 |
| 50 | 49 | Cheyne, for Sick & Incurable Chldn | 29 | ... | 5,829 | 2,938 | 1,773 | 610 | 5,321 | 64 |
| 22 | 20 | Kensington, General ... ... | 255 | 19,364 | 3,439 | 2,066 | 71 | 1,521 | 3,658 | 325 |
| 11 | 9 | Kensington, for Children .. | 152 | 8,511 | 1,391 | 2,078 | 209 | ... | 2,287 | 200 |
| 46 | 31 | Paddington Green, for Children | 677 | 41,202 | 8,473 | 4,104 | 1,287 | 511 | 5,902 | 185 |
| 104 | 72 | Victoria, for Children ... ... | 1,088 | 57,754 | 11,756 | 8,748 | 2,969 | 507 | 12,224 | 2,002 |
| 75 | 60 | Chelsea, for Women ... ... | 647 | 7,162 | 11,010 | 5,989 | 1,657 | 5,415 | 13,061 | 255 |
| 127 | 97 | Cancer ... ... ... ... | 636 | 29,350 | 26,175 | 2,828 | 9,949 | ... | 12,777 | 21,060 |
| 162 | 135 | Female Lock ... ... ... | 633 | ... | 12,634 | 4,996 | 749 | 4,631 | 10,376 | 442 |
| 24 | 23 | Banstead Surgical Home ... | 103 | ... | 1,010 | 346 | 92 | 608 | 1,046 | ... |
| 35 | 25 | Acton Cottage ... ... ... | 286 | 11,214 | 2,348 | 2,001 | 189 | 782 | 2,972 | ... |
| 70 | 40 | Ealing Cottage ... ... ... | 606 | ... | 4,423 | 2,791 | 627 | 2,411 | 5,829 | 6,654 |
| 18 | 11 | Epsom and Ewell Cottage ... | 195 | ... | 1,780 | 1,102 | 139 | 380 | 1,621 | 300 |
| 27 | 21 | Hounslow Cottage ... ... | 247 | 3,184 | 2,185 | 1,569 | 430 | 848 | 2,847 | 343 |
| 40 | 31 | Reigate and Redhill Cottage ... | 643 | ... | 3,641 | 2,512 | 299 | 304 | 3,115 | ... |
| 21 | 12 | Surbiton Cottage ... ... | 276 | ... | 1,338 | 1,246 | 391 | 257 | 1,894 | 590 |
| 24 | 19 | Teddington Cottage ... ... | 242 | ... | 1,434 | 1,567 | 239 | 123 | 1,929 | 655 |
| 42 | 33 | Wimbledon ... ... ... | 474 | ... | 2,755 | 1,497 | 470 | 1,571 | 3,538 | ... |
| 35 | 30 | Wimbledon (Nelson) ... ... | 505 | 2,400 | 2,762 | 1,515 | 308 | 887 | 2,710 | ... |
| 35 | 24 | St. Luke's ... ... ... | 185 | ... | 2,448 | 1,623 | 499 | 432 | 2,554 | 100 |
| 2,134 | 1,748 | | 17,042 | 448,557 | 278,574 | 107,007 | 54,646 | 73,394 | 235,047 | 80,594 |
| | | DISPENSARIES. | | | | | | | | |
| ... | ... | Kilburn, Maida Vale ... ... | ... | 5,441 | 395 | 453 | 40 | ... | 493 | ... |
| ... | ... | Kilburn Provident ... ... | ... | 11,656 | 1,060 | 87 | 20 | 963 | 1,070 | ... |
| ... | ... | Notting Hill Provident ... ... | ... | 7,346 | 436 | 77 | 26 | 359 | 462 | ... |
| ... | ... | Royal Pimlico Provident... ... | ... | 5,340 | 323 | 205 | 30 | 40 | 275 | ... |
| 2,134 | 1,748 | | 17,042 | 478,340 | 280,788 | 107,829 | 54,762 | 74,756 | 237,347 | 80,594 |

### NEWINGTON AND SOUTH DISTRICT.

**Comprising Battersea, Wandsworth, Tooting, Balham, Streatham, Brixton, Lambeth, Newington, Southwark, Bermondsey, Camberwell, Greenwich, Deptford, Lewisham, Blackheath, Woolwich, &c.**

| No. of Beds. | No. of Beds Daily Occupied. | Hospitals. | In-patients. | Out-patient Attendances. | Total Expenditure. | Income. | | | Total Income. | Legacies not included in preceding column. |
|---|---|---|---|---|---|---|---|---|---|---|
| | | | | | | Charitable. | Proprietary. | Patients' Payments. | | |
| | | | | | £ | £ | £ | £ | £ | £ |
| 643 | 539 | Guy's ... ... ... ... | 7,804 | 375,936 | 111,336 | 25,937 | 59,889 | 6,601 | 92,427 | 21,233 |
| 667 | 591 | King's College ... ... ... | 7,756 | 118,541 | 93,186 | 20,851 | 15,868 | 42,068 | 78,787 | 8,399 |
| 18 | 9 | Phillips' Memorial Homœopathic | 151 | 2,276 | 1,551 | 511 | 378 | 535 | 1,424 | ... |
| 76 | 70 | Miller ... ... ... ... | 1,100 | 124,404 | 15,919 | 10,914 | 2,843 | 1,469 | 15,226 | 614 |
| 68 | 53 | St. John's, Lewisham ... ... | 487 | 20,143 | 7,247 | 3,309 | 900 | 3,424 | 7,633 | 25 |
| 1,014 | 851 | St. Thomas's ... ... ... | 9,234 | 271,849 | 148,941 | 4,782 | 69,483 | 47,982 | 122,247 | 586 |
| 395 | 325 | Seamen's ... ... ... ... | 3,630 | 59,940 | 39,445 | 23,330 | 7,738 | 19,211 | 50,279 | 1,175 |
| 60 | 52 | Bolingbroke Hospital ... ... | 754 | 28,276 | 9,263 | 5,074 | 1,137 | 1,892 | 8,103 | ... |
| 76 | 41 | Evelina, for Children ... ... | 839 | 32,370 | 9,741 | 2,786 | 4,410 | 259 | 7,455 | 750 |
| 60 | 38 | Home for Sick Children... ... | 519 | 7,106 | 8,359 | 2,549 | 218 | 618 | 3,385 | 195 |
| 14 | 10 | British Home for Mothers and Babies ... ... ... ... | 188 | 3,900 | 1,944 | 639 | 598 | 754 | 1,991 | ... |
| 36 | 33 | General Lying-In... ... ... | 914 | 15,711 | 9,028 | 4,462 | 3,155 | 373 | 7,990 | ... |
| 50 | 34 | Clapham Maternity & Dispensary | 826 | 3,816 | 3,911 | 1,115 | 720 | 2,610 | 4,445 | ... |
| 80 | 72 | South London, for Women ... | 1,106 | 31,416 | 12,623 | 4,171 | 2,712 | 4,177 | 11,060 | 125 |
| 106 | 69 | Royal Waterloo ... ... ... | 811 | 21,068 | 11,062 | 8,473 | 3,005 | 3 | 11,481 | 761 |
| 42 | 34 | Royal Eye ... ... ... ... | 516 | 56,513 | 6,810 | 2,520 | 1,316 | 2,983 | 6,819 | 970 |
| 28 | 11 | Beckenham Cottage ... ... | 243 | 1,623 | 1,441 | 1,337 | 82 | 491 | 1,910 | ... |
| 26 | 19 | Blackheath Cottage ... ... | 219 | 1,022 | 2,145 | 1,497 | 893 | 292 | 2,682 | ... |
| 42 | 12 | Bromley Cottage ... ... ... | 287 | 205 | 2,090 | 1,130 | 775 | 478 | 2,383 | ... |
| 27 | 20 | Chislehurst, &c., Cottage ... | 308 | ... | 2,457 | 1,229 | 295 | 648 | 2,172 | ... |
| 22 | 15 | Eltham Cottage ... ... ... | 240 | ... | 1,355 | 956 | 261 | 470 | 1,687 | 50 |
| 12 | 6 | Sidcup Cottage ... ... ... | 110 | 90 | 975 | 535 | 73 | 265 | 873 | ... |
| 34 | 25 | Livingstone Cottage ... ... | 412 | ... | 2,269 | 1,823 | 133 | 160 | 2,116 | ... |
| 23 | 17 | Victoria Hospital, Kingston ... | 376 | ... | 1,586 | 1,135 | 301 | 384 | 1,820 | 90 |
| 3,619 | 2,946 | | 38,830 | 1,176,205 | 504,684 | 131,065 | 177,183 | 138,147 | 446,395 | 34,973 |
| | | DISPENSARIES. | | | | | | | | |
| — | — | Battersea Provident ... ... | ... | 80,000 | 3,255 | 160 | 56 | 3,040 | 3,256 | ... |
| — | — | Brixton, &c. ... ... ... | ... | 9,743 | 1,020 | 256 | 686 | 40 | 982 | 200 |
| — | — | Camberwell Provident ... ... | ... | 48,991 | 861 | 182 | 179 | 452 | 813 | ... |
| — | — | Clapham ... ... ... ... | ... | 7,821 | 508 | 218 | 140 | 211 | 569 | ... |
| — | — | East Dulwich Provident ... | ... | 12,943 | 1,013 | 88 | 28 | 922 | 1,038 | ... |
| ... | — | Forest Hill Provident ... ... | ... | 8,428 | 518 | 164 | 22 | 326 | 512 | ... |
| — | — | Greenwich Provident ... ... | ... | 18,085 | 444 | 44 | 11 | 405 | 460 | ... |
| — | — | Wandsworth Common ... ... | ... | 926 | 173 | 12 | 20 | 141 | 173 | ... |
| ... | — | Woolwich, &c., Provident ... | ... | 10,161 | 598 | 42 | ... | 499 | 541 | ... |
| 3,619 | 2,946 | | 38,830 | 1,373,303 | 513,074 | 132,231 | 178,325 | 144,183 | 454,739 | 35,173 |

### THE MEDICAL CHARITIES OF LONDON.—A SUMMARY OF THE WORK DONE IN 1918.

It will be seen from the following summary that *One hundred and forty-eight thousand three hundred and thirty-one* patients were admitted into the Voluntary Hospitals of London during the year 1918, and that the attendances in the Out-Patient Departments and Dispensaries numbered *Five millions and seventy-nine thousand nine hundred and twenty* at a cost of *£2,127,462*. The Ordinary Income amounted to *£1,999,004*, leaving a deficiency of *£128,458* on the year's work. The Legacies received in 1918 amounted to *£274,155*.

| No. of Beds. | No. of Beds Daily Occupied. | Hospitals and Dispensaries. | In-patients. | Out-patient Attendances. | Total Expenditure. | Income. | | | Total Income. | Legacies not included in preceding column. |
|---|---|---|---|---|---|---|---|---|---|---|
| | | | | | | Charitable. | Proprietary. | Patients' Payments. | | |
| | | | | | £ | £ | £ | £ | £ | £ |
| 3,619 | 2,946 | Newington and South District... | 38,830 | 1,373,303 | 513,074 | 132,231 | 178,325 | 144,183 | 454,739 | 35,173 |
| 1,847 | 1,471 | City and East Central District... | 19,774 | 780,652 | 298,170 | 92,064 | 98,293 | 73,876 | 264,233 | 17,275 |
| 1,084 | 893 | Westminster District ... ... | 10,047 | 441,250 | 166,426 | 102,904 | 20,774 | 55,918 | 179,596 | 33,723 |
| 2,102 | 1,765 | St. Marylebone, &c., District ... | 19,733 | 655,133 | 300,787 | 125,571 | 54,846 | 93,697 | 274,114 | 49,781 |
| 2,134 | 1,748 | Kensington and West District ... | 17,042 | 478,340 | 280,788 | 107,829 | 54,762 | 74,756 | 237,347 | 80,594 |
| 1,937 | 1,607 | Islington & North-West District | 16,680 | 559,556 | 236,751 | 106,253 | 27,594 | 94,035 | 227,882 | 24,166 |
| 1,837 | 1,558 | Stratford and East-End District | 26,225 | 791,686 | 331,466 | 257,453 | 57,105 | 46,535 | 361,093 | 33,443 |
| 14,560 | 11,988 | | 148,331 | 5,079,920 | 2,127,462 | 924,305 | 491,699 | 583,000 | 1,999,004 | 274,155 |

